# Hemostasis-On-a-Chip: Impedance Spectroscopy Meets Microfluidics for Hemostasis Evaluation

**DOI:** 10.3390/mi10080534

**Published:** 2019-08-14

**Authors:** Shadi Karimi, Josep Farré-Lladós, Enrique Mir, Ginés Escolar, Jasmina Casals-Terré

**Affiliations:** 1 Mechanical Engineering Department–MicroTech Lab., Universitat Politècnica de Catalunya, Colom 7-11, 08222 Terrassa, Spain; 2 Instituto de Investigación contra la Leucemia Josep Carreras, Muntaner 383, 08021 Barcelona, Spain; 3 Servicio de Hemoterapia y Hemostasia, Hospital Clínic de Barcelona, Instituto de Investigaciones Biomédica August Pi i Sunyer (IDIBAPS), Universidad de Barcelona, 08007 Barcelona, Spain

**Keywords:** organ-on-a-chip, vein-on-a-chip, impedance, microfluidics, hemostasis

## Abstract

In the case of vascular injury, a complex process (of clotting) starts, involving mainly platelets and coagulation factors. This process in healthy humans is known as hemostasis, but when it is deregulated (thrombosis), it can be the cause of important cardiovascular diseases. Nowadays, the aging of the population and unhealthy lifestyles increase the impact of thrombosis, and therefore there is a need for tools to provide a better understanding of the hemostasis mechanisms, as well as more cost-effective diagnosis and control devices. This study proposes a novel microflow chamber, with interchangeable biomimetic surfaces to evaluate global hemostasis, using reduced amounts of blood sample and reagents, and also a minimized time required to do the test. To validate the performance of this novel device, a study on the new oral anticoagulant Apixaban (APIX) has been performed and compared to previous conventional techniques. The test shows an excellent agreement, while the amount of the required sample has been reduced (only 100 µL is used), and the amount of reagent as well. An imprinted electrode embedded in the chamber in order to measure the impedance during the coagulation process. This approach distinguishes the impedance behavior of plasma poor in platelets (PPP) and plasma rich in platelets (PRP) for the first time.

## 1. Introduction

Hemostasis is the result of the collaboration between plasma and blood cells to stop bleeding during the initial steps of wound healing. Current unhealthy habits and aging of the population alter the physiologic balance of blood coagulation, resulting in thrombotic complications. During thrombosis, the aggregation of platelets and coagulation products can prevent blood flow and cause damage in downstream organs, resulting in ischemia and/or tissue death.

Evaluation of hemostasis requires a combination of routine and specialized tests to assess the interaction of platelet and fibrin components involved in blood clotting. Evaluation of hemorrhagic disorders does also require time-consuming tests and sophisticated equipment to identify the altered functional pathways. The diagnosis of thrombotic disorders implies the analysis of the presence and function of activators and inhibitors participating in these coagulation mechanisms.

Nowadays, the evaluation of hemostasis is performed under static conditions. The introduction of microfluidics approaches provides the possibility to mimic blood flow and ex vivo coagulation with minute volume samples, therefore it is already being used as an important tool to improve the knowledge on hemostasis processes [1,2,3].

A common limitation of the majority of current tests applied to the evaluation of hemostasis is that they are performed under static conditions, on samples of plasma or enriched platelet suspensions [4]. In contrast with these static tests, bleeding or thrombotic complications occur in whole blood flowing through damaged vessels. Thus, current tests applied to the evaluation of hemostasis provide a fragmented view of the isolated components of the hemostasis, disregarding the interactions that must necessarily occur in flowing blood. Studies using perfusion annular and parallel chamber technologies with circulating blood have contributed significantly to the knowledge on the function of platelets in the hemostatic mechanism and the thrombotic complications under shear conditions [5,6]. More recently developed microfluidic devices have facilitated the implementation of perfusion assays in a more simplified way than the classic approaches, using small blood samples and facilitating the evaluation of the results [1,2,3,7].

Therefore, the study of blood flow biorheology is of great interest for a better understanding of hemostasis and the effects of antithrombotic drugs [8,9]. Now microfluidics and micro biomimetic flow chambers can provide platforms for the ex vivo study of the effects of flow upon blood coagulation and fibrin formation [10,11,12].

Due to the introduction of direct oral anticoagulants (DOACs) to circumvent the frequent monitoring and dose adjustment with classic Vitamin K antagonists (VKAs), there is now a need for methods to measure the anticoagulant effects of these drugs in several situations: Hospitalized or critically ill or bleeding patients. Uncertainties about the use of DOACs in patients requiring an urgent invasive procedure, suspicion of overdose, recurrence of thrombotic events or confirmation of adherence, need to be addressed [13,14]. Assessment of the impact of anticoagulant therapies is simple for VKAs, but very complex for the DOACs. Monitoring antiplatelet therapy or assessing the potential risk of bleeding or thrombosis requires specialized equipment, specific devices and a definition of cut-off values for each drug. Unfortunately, tests used to assess the effects of DOACs on coagulation are drug-specific, and not routinely available at clinical labs. Availability of reliable point of care (POC) tests which require less volume of sample and a short turnaround of results would facilitate the evaluation of the anticoagulant activity, the identification of specific patient groups and the guidance of reversal agents in case of overdose.

Moreover, antiplatelet agents are prescribed and administered at fixed doses to patients at risk of cardiovascular complications. Although regular monitoring of their actions is not advised, several studies have demonstrated that responses to antiplatelet drugs are not uniform [15]. There are subgroups of patients in whom different laboratory tests indicate suboptimal responses to antiplatelet drugs. This condition, initially defined as “resistance” to the antiplatelet agent, has evolved to a more descriptive concept of patients with “high on-treatment platelet reactivity” (HPR) [16]. HPR exposes patients to an increased risk of major adverse cardiovascular events, and may require dual therapy. Conversely, the concept “low on-treatment platelet reactivity” (LPR) defines subgroups of patients exposed to an enhanced bleeding risk. Optimized antiplatelet therapy based upon a reliable functional assay will improve the balance of efficacy vs. safety in subgroups of patients.

The combination of techniques evaluating the platelet and coagulation elements of hemostasis is the initial step towards the development of POC devices that could efficiently and reliably evaluate the contribution of both components in minute blood samples or at patients’ bedsides, which is highly desirable.

The research work presented here leads to a fundamental contribution to the understanding of hemostasis processes inside vessels, which is of utmost importance to the medical community in order to enhance the treatment of cardiovascular diseases. The design and the validation of the microflow chamber as a tool to characterize a dominant behavior of plasma poor in platelets (PPP) or plasma rich in platelets (PRP) is the base for a highly reliable point-of-care device for antithrombotic treatment monitoring. In the past few years, direct oral anticoagulants (DOACs) were introduced to circumvent the frequent monitoring of classical ones, and therefore decrease the burden on public health systems in countries where the growth of the elderly population has spread the cardiovascular disease impact. 

But there is a need to measure the effectiveness of these treatments, especially near the patient. The use of biomimetic microfluidic channels for studying the hemostasis process reduces the time and the number of samples required, and provides a tool to provide results near the patient.

## 2. Materials and Methods

### 2.1. Microflow Chamber Design and Manufacturing

**Figure 1:**
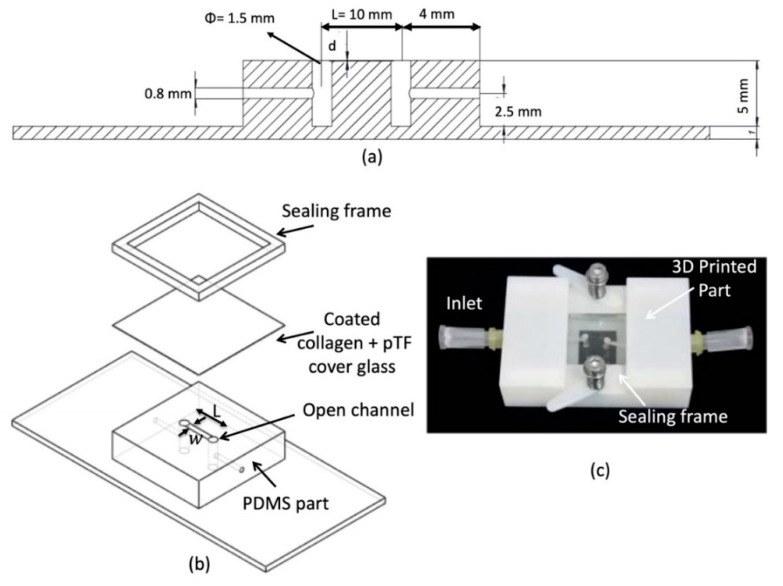
3D schematics and the picture of the biomimetic microfluidic channel. (**a**) the cross-sectional view of the microfluidic channel. (**b**) 3D assembly of the different parts of the microfluidic channel. (**c**) Real image of the microfluidic channel.

The microfluidic device has three main parts: A polydimethylsiloxane (PDMS) part that contains an open microfluidic channel, and it has been manufactured by conventional lithography methods, with a glass-cover to mimic the vasculature tissue and a 3D-printed frame, see Figure 1b. The goal of the printed frame is to apply pressure on the glass in order to seal the channel. Two springs that are embedded in the frame (Figure 1c) allow the exchange of the glass to morphologically characterize the thrombi after the tests.

As shown in Figure 1 the microfluidic PDMS part has a channel (width (w = 500 µm), length (L = 10 mm), depth (d = 50 µm)). Two side channels are made with a 1.5 mm puncher to connect with the inlet and outlet of the device. Finally, the channel is sealed with the glass, which is previously coated with collagen and placenta tissue factor to mimic blood vessel structure.

### 2.2. Biomimetic Coatings and Sample Preparation

Glass slides (18 mm × 18 mm × 1 mm, from Delta Lab) and interdigitated electrodes (Micrux ED_IDE3-Au) were cleaned and functionalized with collagen and tissue factor. Functionalized slides or electrodes were stored at 4 °C overnight. Once assembled, channels were coated overnight with collagen Type I (Chronology Corp. Havertown, PA, USA) and tissue factor (Innovin, Siemens, Madrid, Spain) to achieve coating concentrations equivalent to 30.9 mg/cm^2^ and 0.95 ng/cm^2^, respectively, as previously described [17], and were flushed with saline prior to perfusion in order to eliminate the remaining collagen over the surface.

Blood samples were collected from healthy adults (n = 10) after written consent in accordance with the ethics committee from the Hospital Clinic de Barcelona.

Blood was drawn into a syringe prefilled with low molecular weight heparin (LMWH) and centrifuged at 14,000 rpm for 2 min to obtain plasma poor in platelets (PPP) and at 1,000 rpm for 3 min to obtain plasma rich in platelets (PRP). PPP and PRP will be used to study the formation of fibrin and platelets aggregates, respectively.

### 2.3. Flow Assays

The study of blood or plasma flow in a square microchannel can be analyzed by solving the steady-state Navier-Stokes equation for low Reynolds numbers:

(1) ∇·u=0.

(2) ∇P=∇·τ

where u is velocity, P is pressure and τ is the wall shear stress.

The wall shear stress can determine the growth rate of the thrombi during the coagulation process, and it is one of the parameters under the study [18]. Blood is stored in a collection tube with anti-coagulants, and then using a centrifuge, the main cells (red blood cells (RBCs) and white blood cells (WBCs)) are separated from the plasma. Plasma constitutes around 55% of the blood volume, and contains numerous proteins, including the clotting factors which are the focus of this study, and other suspended materials. For blood coagulation studies, PRP and PPP are used to evaluate the function of platelets and fibrin(ogen), respectively. While whole blood behaves as a non-Newtonian fluid, and the viscosity changes with the applied shear rate, plasma with a water content of almost 95% behaves as a Newtonian Fluid.

For Newtonian fluids, the shear stress is linearly proportional to the shear rate $\dot{\gamma }$, and the shear rate tensor can be expressed as $\bar{\bar{\tau }}=\eta \cdot \bar{\bar{\dot{\gamma }}}$, being that η is the viscosity of the fluid. Using the relation between the flow rate Q in a rectangular microchannel and the Pressure loss [19]

(3) $$Q\approx \frac{wd^{3}}{12\eta L}\nabla P\left[1-0.63\frac{d}{w}\right]$$

where *w* is the width of the channel, *d* is the depth and *L* the length.

Then the wall shear rate $\dot{\gamma }$ can be related to the volumetric flow rate Q according to [20]:

(4) $$\dot{\gamma }\approx \frac{32\;Q}{\pi \;D_{h}^{3}}$$

where $D_{h}=2w\cdot d/\left(w+d\right)$ is the hydraulic diameter of the rectangular channel. Since plasma behaves approximately as a Newtonian fluid, a wall shear rate $\dot{\gamma }$ of 300 s^−^^1^ was achieved on the glass surface, applying a flow rate of 0.1 mL/h to the microchannel through a syringe pump.

### 2.4. Image Capture and Analysis

The perfused channels were fixed with paraformaldehyde 1% for 15 min at 4 °C and further incubated with glycine 1% for 10 min to reduce high background staining due to free unreactive aldehyde groups. Then the channels were blocked with 1% bovine serum albumin (BSA) for 15 min prior to incubating with specific antibodies. A combination of indirect and direct immune-fluorescence was carried out as follows.

First, platelets were stained with a mouse anti-CD36 primary antibody for 1 h at room temperature (RT, 37 °C) in a humidified chamber. Then, a secondary antibody anti-mouse Alexa Fluor 488 was incubated together with a conjugated antibody anti-fibrinogen Alexa Fluor 594 for 1 h at RT in a humidified chamber. Pictures were acquired using a confocal microscope (SP5, Leica Microsystems, Barcelona, Spain). Acquired images from the channels of the micro-chamber were analyzed using the ImageJ software (v 1.43m), (Rasband, W.S., ImageJ, National Institutes of Health, Bethesda, MD, USA). 

Co-distributions between the platelets (green) and fibrin (red) were analyzed. The intensity of each marker was densitometrically analyzed individually in each picture, superposed and expressed as a percentage of the covered surface corresponding to the entire image.

### 2.5. Impedance Characterization

**Figure 2:**
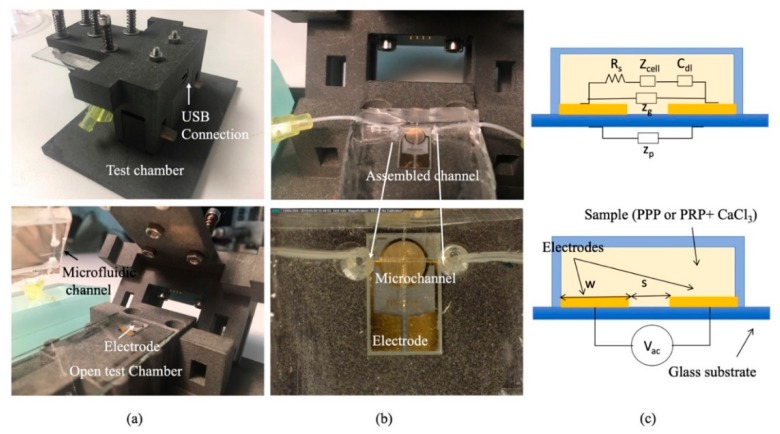
(**a**) Pictures of the test chamber assembled and open. (**b**) Pictures of the microfluidic channel inside the test chamber with the thrombogenic surface with embedded electrodes. (**c**) Impedance model of the electrode inside the channel.

Electrochemical Impedance Spectroscopy (EIS) studies the system response to a small amplitude sinusoidal signal at different frequencies, and it can give information about the analytic molecules in a fluidic solution (for instance, a blood sample). In this new chamber, the glass slide was replaced by the glass with printed electrodes (ED_IDE3-Au) from Micrux (Oviedo, Spain), see Figure 2.

Figure 2a,b show different images of the biomimetic microfluidic channel for impedance measurements. Now the glass cover of the channel has the electrodes (ED_IDE3-Au) from Micrux which are 180 gold strips separated 5 µm apart and with 5 µm in width, forming a 3.5 mm circle. The electrodes close the channel and are biomimetically covered, as described in Section 2.2. Figure 2c portrays the schematics of the electric equivalent circuit of the electrodes. When the plasma with the platelets (PRP—plasma rich in platelets) or fibrinogen (PPP—plasma poor in platelets) flows on the surface of the electrodes, the sensor can be used to determine the composition of the fluidic solution, since their components show a different imaginary and real impedance response at different frequencies. For an arbitrary electrode, its impedance (Z_E_) can be described by different components according to [21]:

(5) $$Z_{E}=\left(R_{s}+2Z_{\mathrm{dl}}\right)\|\left(\frac{1}{{j\omega C}_{g}}\right)\|Z_{p}$$

where R_s_ is the resistance of the solution, $Z_{\mathrm{dl}}=\left(R_{\mathrm{ct}}+Z_{w}\right)\|\left(\frac{1}{{j\omega C}_{\mathrm{dl}}}\right)$ is the double-layer impedance and C_g_ is the dielectric capacitance and Z_p_ the parasitic capacitance of the substrate.

The resistance of the solution, $R_{s}=g/\left(\sigma W\right),$ relates to the geometry of the electrode g=g(L,S), where L is the length of the electrode and S the separation between electrodes and the conductivity of the solution σ. In our design, W, the width of the electrode in-plane, and g, are constant parameters of the electrode. 

Therefore, R_s_ is related to the conductivity of the solution, $\sigma ={\mathrm{qn}}_{i}\;\left(\mu_{p}+\mu_{n}\right)$, where q is the electric charge, $\mu_{p}$ and $\mu_{n}$ are the ionic mobilities of the dominant positive and negative ions in the solution, and $n_{i}$ is the ionic concentration, which can vary during coagulation.

The double-layer impedance ($Z_{\mathrm{dl}}$) captures the phenomena around the electrode; the term $R_{\mathrm{ct}}$ captures the charge transfer between electrodes and $Z_{w}$ the mass-transfer between them. In case of non-faradaic electrodes, since there is no surface reactions (Rct→∞), therefore, there is no mass transfer. Besides, $Z_{w}\approx 0$, $Z_{\mathrm{dl}}=\left(\frac{1}{{j\omega C}_{\mathrm{dl}}}\right)$, where C_dl_ originates from the adsorbed charge layer and diffuse layer charge. For electrode separation higher than Debye length (λ∼1 μm), C_dl_ can be described by diffuse layer capacitance $C_{dl}=C_{dif}=A\sqrt{\frac{2\varepsilon n_{i}q^{2}}{kT}}\cosh\left(\frac{q\;V_{ac}}{2kT}\right)$, where A is the area of the electrode (A = wL), V_ac_ is the voltage applied, q is the electric charge, k is the Boltzmann constant, T is the temperature of the solution, and ε is the permittivity of the medium separating the electrodes.

For a parallel plate system, the geometric capacitance form by the electrodes and the solution is $C_{g}=A\;\varepsilon /S$, where A is the area of the electrodes, ε the permittivity of the medium separating the electrodes and S is the separation between the electrodes.

If the substrate is highly resistive, such as glass (low dielectric constant), the frequency response of an ideal non-Faradaic shows three different regions:

1. For low frequencies $f_{\mathrm{low}}=\frac{2}{2{\pi R}_{s}C_{\mathrm{dl}}}$, $C_{\mathrm{dl}}$ dominates the impedance measured.
2. For frequencies $f_{\mathrm{low}}<f<f_{\mathrm{high}}=\frac{2}{2{\pi R}_{s}C_{g}}$, $R_{s}$ is the dominant impedance.
3. For frequencies $f>f_{\mathrm{high}}$, $C_{g}$ is the dominant impedance.

The time dependence of the different components is:

R_s_ is depending upon the concentration of the ions in the solution, since during coagulation different ions are involved. R_s_ will change during the coagulation process; basically, if the concentration is increased, the conductivity will increase and R_s_ decreases.

C_dl_ will also increase if the concentration of ions is increased, and finally, the C_g_ is independent on the ion concentration. C_g_ changes could be related to changes in the permittivity of the solution, that could change if there were volume changes of the sample, but since our system is inflow, the volume covering the electrodes is constant.

Therefore, as the blood clot is forming, the change in ion species will be detectable using impedance, and the combination of this quantification method with the biomimetic microfluidic chamber allows a quantification of the effects of the shear rate of the process of clot formation.

To study this change of impedance, PalmSens 4 EIS was connected to the electrodes to measure impedance at different frequencies, see USB connection in Figure 2. A 100 µL PPP or PRP sample is loaded to an Eppendorf and withdrawn with a syringe pump.

Prior to each test, the plasma (PPP or PRP) was placed at room temperature for 30 min. Then, the plasma sample was mixed with 1 µL calcium chloride (CaCl_2_ (5mM)) to induce coagulation. Electrical impedance across the electrodes between 10 Hz and 1 MHz was measured while a sinusoidal voltage of 250 mV was applied.

Illustration of the electrodes in contact with the sample and a photograph of the microfluidic chip is shown in Figure 2.

## 3. Results

### 3.1. Flow Assay Results

**Figure 3:**
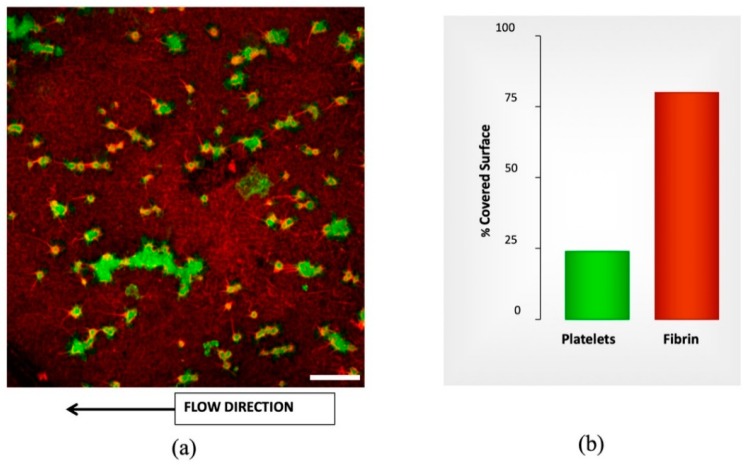
Confocal image labeled by immunofluorescence for morphometric analysis from microfluidic studies. (**a**) Confocal image showing platelets labeled byanti-CD36 Alexa Fluor 488 and Fibrin labeled by anti-fibrin(ogen) Alexa Fluor 594. The thrombogenic surface is a biomimetic combination with type-I fibrillar collagen (30.9 mg/cm^2^) and tissue factor (0.95 ng/cm^2^). Scale bar = 20 µm. (**b**) The plot shows the quantification of platelet aggregates (green) and fibrin masses (red) interacting with the collagen/tissue factor surface. The bar graphs in the right panel summarize the results as percentages of the total surface exposed.

Figure 3 provides a representative image of the results of microfluidic studies of whole blood with different doses of APLIX and the generated distribution of platelets and fibrin on the perfused surface. The right panel shows bar diagrams representing the proportions of platelet aggregates in green and fibrin masses in red interacting with the collagen/tissue factor surface, as evaluated following the procedure mentioned in Section 2.4. To evaluate the effects of APIX samples with different APIX doses where tested, see Table 1. Bar graphs in the right of Figure 3 quantify the percentages of the total surface exposed that are covered platelets and fibrin platelet, respectively.

Incubations with different APIX doses caused dose-dependent decreases in platelet and fibrin surface, reaching levels of statistical significance at 160 ng/mL (* *p* < 0.05 vs. control without APIX and # *p* < 0.05 vs. APIX 10 ng/mL), see Table 1. Microfluidics studies with recalcified citrated blood show similar results with conventional methods at the same shear rate. Table 1 summarizes the percentages of the covered surface.

### 3.2. Impedance Spectroscopy Assay Results

**Figure 4:**
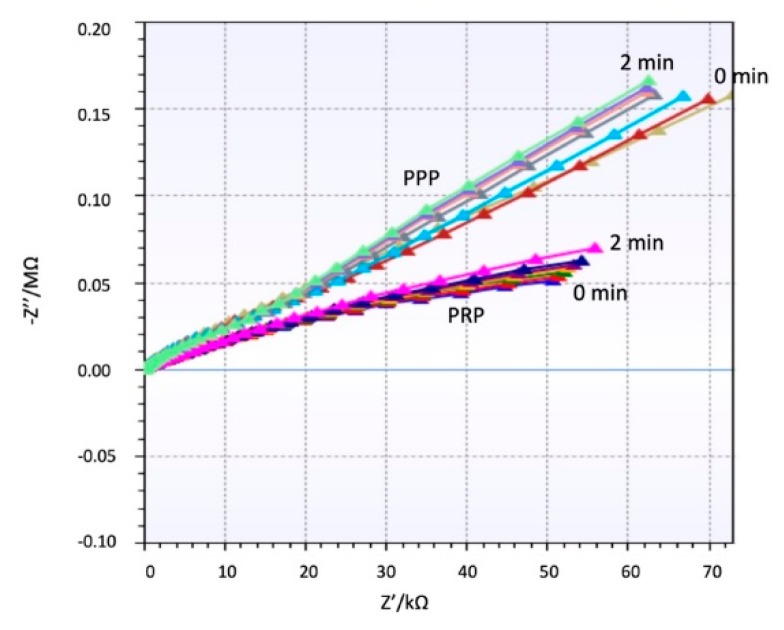
Real and Imaginary part of Impedance for plasma poor in platelets (PPP) and plasma rich in platelets (PRP) over time.

The impedance behavior of the clotting process on PRP and PPP that was studied using a 100 µL of the sample flowing on the coated electrode as follows is differentiated and repeatable. Figure 4 shows a different trend for both samples PRP and PPP. Supplementary material shows different repetitions of the same experiment with other samples and the same behavior. Since PPP creates a complete coverage on the electrodes, it affects equally the real and imaginary parts of the impedance detected. PRP contains a high concentration of platelets creating aggregates during coagulation, see Figure 3 (green dots). Therefore, the main change is focalized on the real part of the impedance.

During clotting the coating of the electrodes is changed, modifying the permittivity layer between the electrodes. In the case of PPP, fibrin formation is uniform on the electrodes, and when the coverage is full, the influence on the solution conductivity changes are reduced. For PRP, the coverage of electrodes is not uniform, since the thrombus is always located on certain points, therefore the changes on conductivity are not relevant; consequently, the variations near the electrode cause a noticeable change on the real part, while the imaginary part slightly changes.

**Figure 5:**
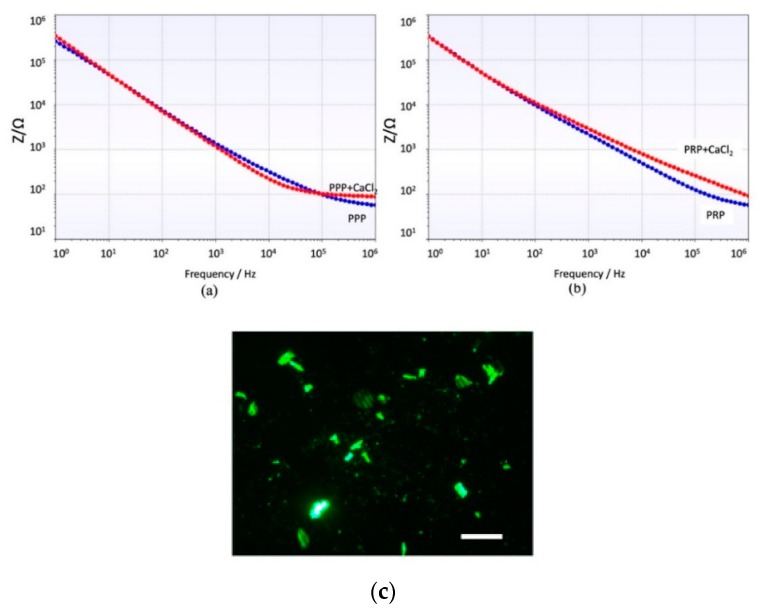
Impedance module at different frequencies after 90 s interaction of the electrode with (**a**) PPP and PPP + CaCl_2_. (**b**) PRP and PRP + CaCl_2_. (**c**) Image of the platelets attached on the electrodes. Scale bar = 10 µm.

The spectrum shows a general capacitive behavior at low frequencies (C_dl_ dominates). The impedance is high at low frequencies and decreases gradually. Around 10^5^ Hz, the impedance decreases to the behavior of becoming more resistive (R_s_ dominates). Both samples PPP and PRP show the same trend, but the impedance of PRP is always higher due to the increased amount of cellular component which increases the resistivity of the solution, especially when CaCl_2_ is added. Figure 5 shows a clear effect of the coagulation on the impedance modulus during the coagulation process, which results in an increase of impedance. This increase is remarkable for low and high frequencies in PRP samples, while in PPP samples the impedance increases at frequencies higher than 10^5^ Hz.

## 4. Discussion

Previous studies proved the importance of studying on hemostasis underflow conditions for a better understanding of the process, and nowadays microfluidics technology provides a perfect platform to study this phenomenon. Most of these studies have focused on a global understanding of whole blood behavior during the coagulation process.

New oral anticoagulants (NOACs) used in patients with more complex treatment cases require close monitoring and assessment of the cross-reaction with classic Vitamin K antagonists (VKAs). Hemostasis is a complex process which at least has a double side contribution from platelets and fibrin(ogen). Conventional evaluation methods use an important amount of reagent and sample, and they are time-consuming. However, the introduction of microfabrication techniques has allowed the replica of vein models and biomimetic surfaces to study the complexity of the coagulation process in biomimetic environments [11,22,23] with a reduced amount of reagents.

These biomimetic approaches still require the use of further biomolecular techniques to quantify the presence and quantity of thrombi or clots. Therefore, the techniques are not compatible with near-patient monitoring strategies. Impedance spectroscopy has successfully been applied to monitor the growth of bacteria colonies in biofilm formation [24,25,26]. The behavior can be extrapolated to the PPP effect that creates a complete coverage on the electrodes similar to biofilm growth, and it affects equally to the real and imaginary part of the impedance detected, as shown in Figure 4.

PRP contains a high concentration of platelets creating aggregates during coagulation, and during this process the electrolytes in solution change and affect the impedance. Previous studies focusing on electrolytes on the solution [27,28] show a different behavior on the real and imaginary part of the impedance, and besides this, a change depending on the concentration of this electrolytes. PRP samples have shown an important influence of the electrolytes in solution and the attachment of the thrombi at the same time, showing a completely differentiated behavior to PPP samples.

The differentiated behavior of PPP and PRP would allow the monitoring of responses to antiplatelet agents present in the samples, while still being able to assess the impact of anticoagulant therapies on the different elements of hemostasis. As for sample volumes, the proposed devices work with a reduced 100 µL sample, and the microfluidics and electronics can be miniaturized in a point of care (POC) system. Besides, the turnaround results are shortened, facilitating a rapid evaluation of the anticoagulant activity, the identification of specific patient groups, and the guidance of reversal agents in case of overdose.

The combination of both analyses (PPP and PRP) in a miniaturized POC device with impedance measurements can evaluate the contribution of platelets and the coagulation mechanism in minute blood samples at patients´ bedsides.

## 5. Conclusions

The present study has combined microfluidics with impedance spectroscopy to diagnose and control coagulation disorders in a biomimetic approach. The proposed device uses a new micro-manufactured microflow chamber with interchangeable biomimetic surfaces to measure coagulation. Evaluation of the impact on coagulation in minute samples (100 µL) spiked with a new oral anticoagulant APIXABAN (APIX) has been performed using this technique and compared to previous conventional techniques. Both tests show an excellent agreement.

The biomimetic surfaces can be imprinted with gold electrodes, adding the capability to quantify the presence of platelets aggregates or fibrinogen formation from a reduced amount of sample and reagents, and also a minimized time for testing.

Different impedance behaviors of plasma poor in platelets (PPP) and plasma rich in platelets (PRP) has been observed. PPP changes during coagulation are more noticeable at higher frequencies and modify both real and imaginary parts of the impedance. PRP changes are remarkable even at low frequencies, but only at the real part of the impedance. This study is the base for a point-of-care device capable of quantifying in a short time and near the patient the effect of anticoagulant on fibrinogen formation or platelet aggregation.

## Figures and Tables

**Table 1:** Percentage of the covered surface at shear rate 600 s^−1^.

[APIX] ng/mL	Platelets	Fibrin
0	23.0 ± 3.0	43.4 ± 4.8
10	17.9 ± 0.9	42.1 ± 1.9
40	14.0 ± 5.3	23.4 ± 7.7
160	5.4 ± 2.2 *#	14.1 ± 4.9 *#

## Supplementary Materials

The following are available online at https://www.mdpi.com/2072-666X/10/8/534/s1, Figures S1 and S2: Real and Imaginary part of Impedance for different samples of PPP and PRP over the time.
