# Supplementary material for: Hemostasis-On-a-Chip: Impedance Spectroscopy Meets Microfluidics for Hemostasis Evaluation
Source: Micromachines (Basel). 2019 Aug 14;10(8):534. doi: 10.3390/mi10080534 (PMC6722990; doi:10.3390/mi10080534)
Supplement: Supplementary file 1 [file micromachines-10-00534-s001.pdf]

Supplementary Materials

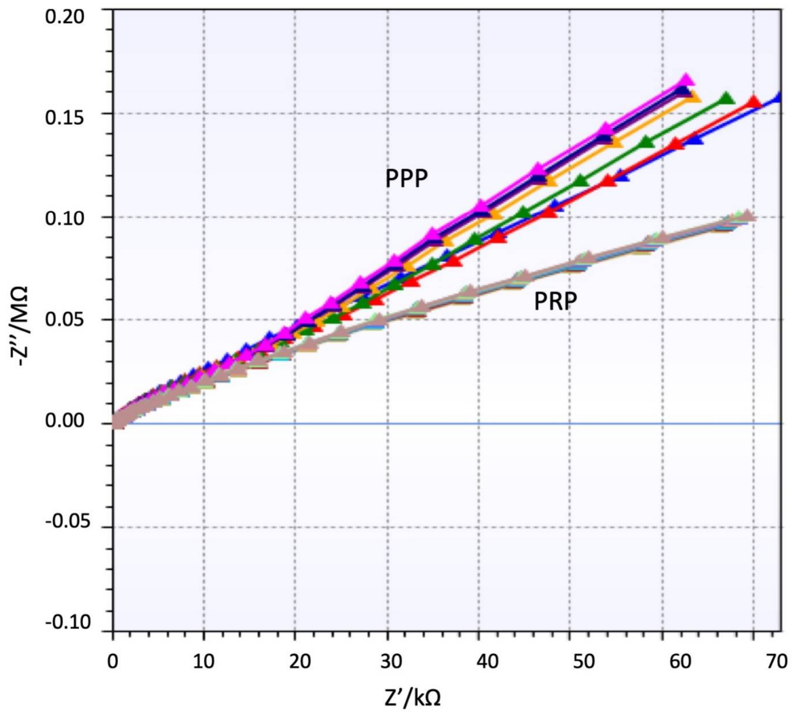

Figure S1. Real and Imaginary part of Impedance for PPP and PRP over the time.

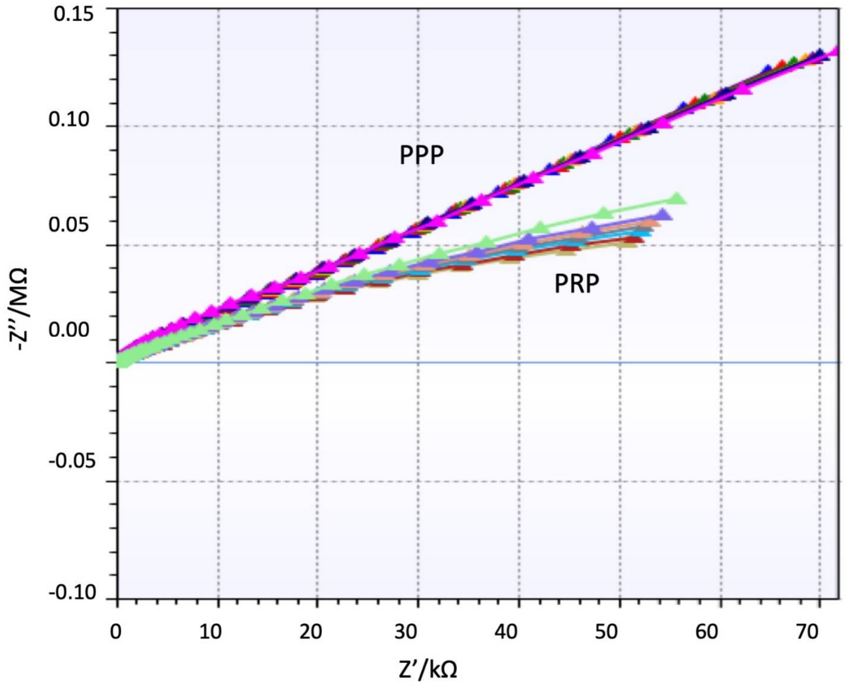

Figure S2. Real and Imaginary part of Impedance for PPP and PRP over the time
